# SARS-CoV-2 Infections and Serologic Responses from a Sample of U.S. Navy Service Members — USS Theodore Roosevelt, April 2020

**DOI:** 10.15585/mmwr.mm6923e4

**Published:** 2020-06-12

**Authors:** Daniel C. Payne, Sarah E. Smith-Jeffcoat, Gosia Nowak, Uzo Chukwuma, Jesse R. Geibe, Robert J. Hawkins, Jeffrey A. Johnson, Natalie J. Thornburg, Jarad Schiffer, Zachary Weiner, Bettina Bankamp, Michael D. Bowen, Adam MacNeil, Monita R. Patel, Eric Deussing, CDC COVID-19 Surge Laboratory Group, Bruce L. Gillingham, CDC COVID-19 Surge Laboratory Group, Rebekah Tiller, Rene Galloway, Shannon Rogers, Brett Whitaker, Ashley Kondas, Peyton Smith, Christopher Lee, James Graziano

**Affiliations:** ^1^CDC; ^2^U.S. Navy.; Division of Viral Diseases, National Center for Immunization and Respiratory Diseases; Division of Viral Diseases, National Center for Immunization and Respiratory Diseases; Division of Viral Diseases, National Center for Immunization and Respiratory Diseases; Division of Viral Diseases; National Center for Immunization and Respiratory Diseases; Division of Viral Diseases, National Center for Immunization and Respiratory Diseases; Division of Viral Diseases, National Center for Immunization and Respiratory Diseases; Division of Viral Diseases, National Center for Immunization and Respiratory Diseases; Division of Viral Diseases, National Center for Immunization and Respiratory Diseases.

Compared with the volume of data on coronavirus disease 2019 (COVID-19) outbreaks among older adults, relatively few data are available concerning COVID-19 in younger, healthy persons in the United States (*1*,*2*). In late March 2020, the aircraft carrier USS Theodore Roosevelt arrived at port in Guam after numerous U.S. service members onboard developed COVID-19. In April, the U.S. Navy and CDC investigated this outbreak, and the demographic, epidemiologic, and laboratory findings among a convenience sample of 382 service members serving aboard the aircraft carrier are reported in this study. The outbreak was characterized by widespread transmission with relatively mild symptoms and asymptomatic infection among this sample of mostly young, healthy adults with close, congregate exposures. Service members who reported taking preventive measures had a lower infection rate than did those who did not report taking these measures (e.g., wearing a face covering, 55.8% versus 80.8%; avoiding common areas, 53.8% versus 67.5%; and observing social distancing, 54.7% versus 70.0%, respectively). The presence of neutralizing antibodies, which represent antibodies that inhibit SARS-CoV-2, among the majority (59.2%) of those with antibody responses is a promising indicator of at least short-term immunity. This report improves the understanding of COVID-19 in the U.S. military and among young adults in congregate settings and reinforces the importance of preventive measures to lower risk for infection in similar environments.

In mid-January, the USS Theodore Roosevelt was deployed to the western Pacific. An outbreak of COVID-19 occurred during deployment, which resulted in the aircraft carrier stopping in Guam at the end of March. During this time, approximately 1,000 service members were determined to be infected with SARS-CoV-2, the virus that causes COVID-19. The United States Navy and CDC investigated this ongoing outbreak during April 20–24; 382 service members voluntarily completed questionnaires and provided serum specimens (a convenience sample comprising 27% of 1,417 service members staying at the base on Guam or on the ship). The 1,417 included persons who were previously infected, currently infected, or never infected. Among these 382 service members, 267 (70%) also provided a nasopharyngeal (NP) swab specimen. Serum specimens were tested for antibody reactivity using a CDC-developed, SARS-CoV-2 spike protein enzyme-linked immunosorbent assay (ELISA) (a pan-immunoglobulin assay) as an indicator of previous SARS-CoV-2 exposure and infection; signal threshold ratio ≥1 was defined as a positive ELISA result (*3*). ELISA-positive specimens were further tested for neutralizing antibodies using a microneutralization assay to detect presence of SARS-CoV-2 inhibiting antibodies (antibody titers >40 defined as positive). Real-time reverse transcription–polymerase chain reaction (RT-PCR) testing of NP swab specimens was used to detect SARS-CoV-2 RNA (*4*). Previous or current SARS-CoV-2 infection was defined as a positive real-time RT-PCR result or positive ELISA result.

At the time of specimen collection, participants completed a questionnaire eliciting information on demographic characteristics, exposure, COVID-19 protective behaviors, health history, and symptoms; participants also reported whether they had had a previous positive COVID-19 test since deployment but before this investigation. Protective behaviors listed on the questionnaire were not mutually exclusive, so participants could select all that applied. Reported symptoms were categorized using the Council of State and Territorial Epidemiologists (CSTE) case definition for COVID-19 (*5*), including category A (at least cough or shortness of breath/difficulty breathing) and category B (no cough or shortness of breath, but two or more other symptoms*) or neither. Demographic, exposure, and symptom characteristics and engagement in protective behaviors were compared among participants infected with SARS-CoV-2 and those having no evidence of previous or current infection, and unadjusted odds ratios (ORs) with 95% confidence intervals (CIs) were calculated. Analyses were performed using SAS statistical software (version 9.4; SAS Institute).

Among the 382 survey participants (Figure 1), 289 (75.7%) were male; their median age was 30 years (interquartile range [IQR] = 24–35 years), 223 (58.4%) were non-Hispanic white, and 28 (7.3%) reported a history of asthma, hypertension, diabetes, or immunosuppression (Table). Among 238 (62.0%) participants with previous or current SARS-CoV-2 infection, 194 (81.5%) reported one or more symptoms, 44 (18.5%) were asymptomatic, and two (0.8%) were hospitalized for COVID-19. Among all participants, the prevalence of previous or current infection among males was higher than that among females (OR = 1.8) but did not differ significantly by age, race, ethnicity, or history of a preexisting medical condition.

**FIGURE 1:**
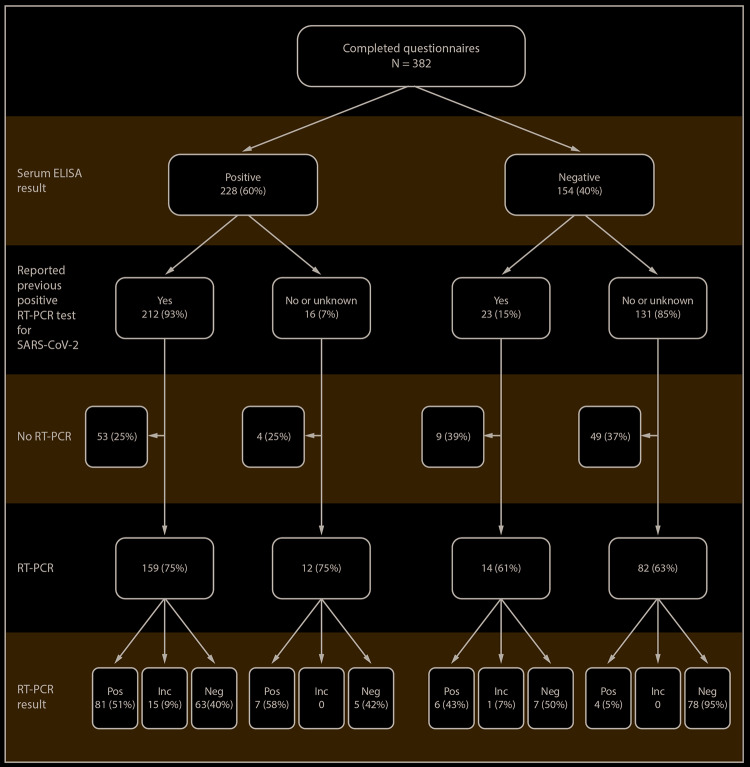
Laboratory results among a convenience sample of U.S. service members who provided serum specimens* (N = 382) and nasopharyngeal swabs (N = 267) for SARS-CoV-2 testing — USS Theodore Roosevelt, April 2020 **Abbreviations:** Ab = antibody; ELISA = enzyme-linked immunosorbent assay; Inc = inconclusive; Neg = negative; Pos = positive; RT-PCR = real-time reverse transcription–polymerase chain reaction. * Of those with positive serum ELISA tests, 59% demonstrated positive microneutralization tests.

**TABLE:** Comparison of U.S. Navy service members with and without previous or current SARS-CoV-2 infection (N = 382) — USS Theodore Roosevelt, April 2020

Characteristic	No. (%)		Infection versus no infection OR (95% CI)^†^
	Current or previous SARS-CoV-2 infection* (N = 238)	No evidence of SARS-CoV-2 infection (N = 144)	
**RT-PCR and antibody results**			
RT-PCR positive and ELISA positive	88 (37.0)	0	N/A
RT-PCR negative and ELISA positive	83 (34.9)	0	N/A
RT-PCR positive and ELISA negative	10 (4.2)	0	N/A
RT-PCR not done and ELISA positive	57 (23.9)	0	N/A
RT-PCR negative or not done and ELISA negative	0	144 (100)	N/A
**Sex**			
Male	190 (65.7)	99 (34.3)	1.80 (1.12–2.89)^§^
Female	48 (51.6)	45 (48.4)	Referent
**Age group (yrs)**			
18–24	77 (68.1)	36 (31.9)	Referent
25–29	50 (64.1)	28 (35.9)	0.84 (0.45–1.54)
30–39	87 (58.8)	61 (41.2)	0.67 (0.40–1.11)
40–59	24 (55.8)	19 (44.2)	0.59 (0.29–1.21)
**Race/Ethnicity^¶^**			
AI/AN or NH/PI	9 (60.0)	6 (40.0)	0.86 (0.29–2.49)
Asian	13 (61.9)	8 (38.1)	0.93 (0.37–2.33)
Black	25 (61.0)	16 (39.0)	0.89 (0.45–1.77)
Hispanic/Latino	47 (61.8)	29 (38.2)	0.92 (0.54–1.58)
Other/Unknown	2 (33.3)	4 (66.7)	0.29 (0.05–1.59)
White	142 (63.7)	81 (36.3)	Referent
**History of asthma, hypertension, diabetes, or immunosuppression**	15 (53.6)	13 (46.4)	0.68 (0.31, 1.47)
**Reported ≥1 symptom**			
Yes	194 (81.5)	90 (62.5)	2.65 (1.65–4.23)^§^
No	44 (18.5)	54 (37.5)	Referent
**Symptoms (among those reporting ≥1 symptom)**			
Symptoms (CSTE criteria)**			
Category A	97 (50.0)	36 (40.0)	3.50 (1.90–6.45)^§^
Category B	67 (34.5)	15 (16.7)	5.81 (2.78–12.11)^§^
Other symptom(s)	30 (15.5)	39 (43.3)	Referent
**Individual symptoms**			
Loss of taste, smell, or both	119 (61.3)	12 (13.3)	10.31 (5.26–20.21)^§^
Palpitations	19 (9.8)	3 (3.3)	3.15 (0.91–10.93)
Fever (documented or subjective)	89 (45.9)	21 (23.3)	2.79 (1.58–4.90)^§^
Chills	85 (43.8)	20 (22.2)	2.73 (1.54–4.84)^§^
Myalgia	109 (56.2)	30 (33.3)	2.56 (1.52–4.32)^§^
Cough	86 (44.3)	29 (32.2)	1.68 (0.99–2.83)
Nausea	40 (20.6)	13 (14.4)	1.54 (0.78–3.05)
Fatigue	107 (55.2)	41 (45.6)	1.47 (0.89–2.43)
Shortness of breath/difficulty breathing	46 (23.7)	17 (18.9)	1.33 (0.72–2.49)
Chest pain	40 (20.6)	15 (16.7)	1.30 (0.68–2.50)
Abdominal pain	39 (20.1)	15 (16.7)	1.26 (0.65–2.42)
Runny nose	108 (55.7)	46 (51.1)	1.20 (0.73–1.98)
Diarrhea	47 (24.2)	20 (22.2)	1.12 (0.62–2.03)
Headache	129 (66.5)	59 (65.6)	1.04 (0.62–1.77)
Vomiting	11 (5.7)	5 (5.6)	1.02 (0.34–3.03)
Sore throat	81 (41.8)	44 (48.9)	0.75 (0.45–1.24)
**Sought medical care for symptoms**	115 (59.3)	35 (38.9)	2.29 (1.37–3.82)^§^
**Hospitalized**	2 (1.0)	0	N/A
**Number of symptoms**			
1–3	51 (26.3)	49 (54.4)	Referent
4–5	37 (19.1)	13 (14.4)	2.74 (1.30–5.75)^§^
6–8	50 (25.8)	16 (17.8)	3.00 (1.51–5.96)^§^
>8	56 (28.9)	12 (13.3)	4.48 (2.15–9.37)^§^
**Still symptomatic at time of survey (n = 275)**			
Yes	65 (34.0)	24 (28.6)	1.29 (0.74–2.26)
No	126 (66.0)	60 (71.4)	Referent
Duration >1 week (n = 186)	70 (55.6)	29 (48.3)	1.34 (0.72–2.47)
**Reported prevention behaviors**			
Increased hand washing	218 (62.1)	133 (37.9)	0.90 (0.42–1.94)
Hand sanitizer use	219 (61.5)	137 (38.5)	0.59 (0.24–1.44)
Avoiding common areas	78 (53.8)	67 (46.2)	0.56 (0.37–0.86)^§^
Face covering use	158 (55.8)	125 (44.2)	0.30 (0.17–0.52)^§^
Increased workspace cleaning	195 (63.5)	112 (36.5)	1.30 (0.78–2.16)
Increased berthing cleaning	156 (61.9)	96 (38.1)	0.95 (0.61–1.47)
Increased distance from others	105 (54.7)	87 (45.3)	0.52 (0.34–0.79)^§^

**Abbreviations:** AI/AN = American Indian or Alaska Native; CI = confidence interval; CSTE = Council of State and Territorial Epidemiologists; ELISA = enzyme-linked immunosorbent assay; N/A = not applicable; NH/PI = Native Hawaiian or other Pacific Islander; OR = odds ratio; RT-PCR = real-time reverse transcription–polymerase chain reaction.

* Current or previous SARS-CoV-2 infection is defined as a positive RT-PCR test result or a reactive antibody result determined by testing performed at CDC laboratories on specimens collected during April 20–24, 2020.

^†^ Odds ratios are unadjusted.

^§^ P-values <0.05 were considered statistically significant.

^¶^ White, black, Asian, AIAN/NHPI, and Other persons were non-Hispanic/Latino. Hispanic/Latino persons might be of any race.

** Category A = ≥1 of cough or shortness of breath/difficulty breathing. Category B = no cough or shortness of breath, but ≥2 of fever, chills, muscle pain, headache, sore throat, no taste or smell disorder.

Among 284 symptomatic participants (194 [68.3%] with previous or current SARS-CoV-2 infections and 90 [31.7%] without), loss of taste (ageusia) or smell (anosmia) were the symptoms most strongly associated with previous or current infection (OR = 10.3), followed by fever (OR = 2.8), chills (OR = 2.7), and myalgia (OR = 2.6) (Figure 2). CSTE-defined category B symptoms were more strongly associated with infection (OR = 5.8) than were category A symptoms (OR = 3.5). Reporting four or more symptoms and seeking medical care for symptoms (OR = 2.3) were significantly associated with infection.

**FIGURE 2:**
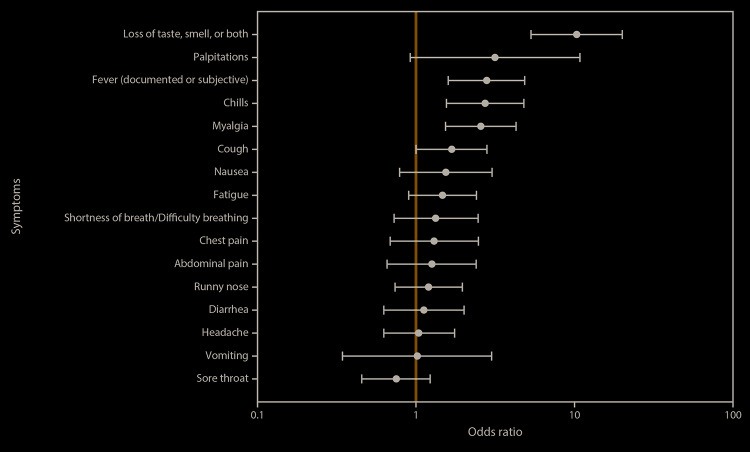
Odds ratios and 95% confidence intervals of previous or current SARS-CoV-2 infection, by individual symptoms among service members reporting at least one symptom (n = 284) — USS Theodore Roosevelt, April 2020

Overall, 228 (59.7%) participants had a positive ELISA result, and among those, 135 (59.2%) also had a positive microneutralization test result. Among those with positive ELISA results, Hispanic/Latino participants were more likely to have positive microneutralization test results (33 of 44; 75.0%) than were participants of non-Hispanic/Latino or unspecified ethnicity (102 of 184; 55.4%) (OR = 2.4; 95% CI = 1.1–5.1). Among the 267 participants who provided an NP swab, 98 (36.7%) had a positive real-time RT-PCR result; 171 (64.0%) persons who provided an NP swab had a positive ELISA result. Among 235 participants who reported a positive SARS-CoV-2 test result before this investigation (defined as during this deployment, mid-January to April 20–24, 2020), 212 (90.2%) had positive ELISA results compared with 16 (10.9%) among 147 not reporting previous positive test results for SARS-CoV-2 (OR = 75.5; 95% CI = 38.5–148.1).

Among 191 symptomatic participants who reported a symptom onset date and had positive real-time RT-PCR results, positive ELISA results, or both, eight had positive real-time RT-PCR and negative ELISA results; for these participants, ≤15 days had elapsed since symptom onset at the time of specimen collection (Figure 3). Among symptomatic participants with positive ELISA results and positive microneutralization test results (n = 107), a median of 22 days (IQR = 15–26) had elapsed since symptom onset at the time of specimen collection (Figure 3). Among 12 participants with positive ELISA results >40 days after symptom onset, eight maintained positive microneutralization test results, including two participants who were tested >3 months after symptom onset.

**FIGURE 3:**
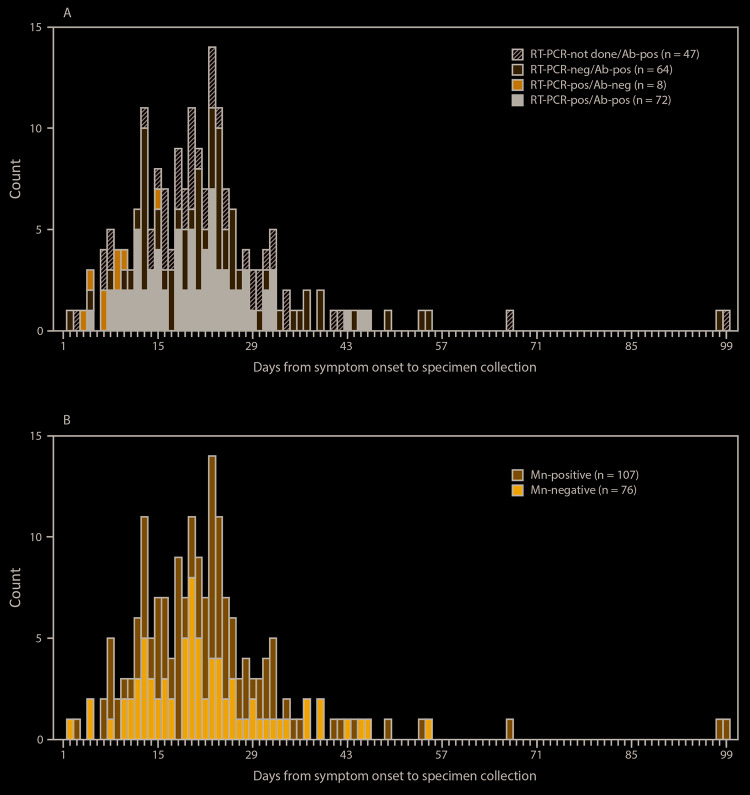
Days from symptom onset* to specimen collection (A) among a convenience sample of participants who had positive real-time reverse transcription–polymerase chain reaction (RT-PCR) or positive enzyme-linked immunosorbent assay (ELISA) test results for SARS-CoV-2 (n = 191) and (B) microneutralization results among those with positive ELISA test results (n = 183) — USS Theodore Roosevelt, April 2020 **Abbreviations:** Ab = pan-immunoglobulin antibody response; Mn = microneutralization test. * Three persons who reported symptoms and had previous or current infection did not report a date of symptom onset and were not included in this figure.

Prevalence of previous or current infection was higher among participants who reported contact with someone known to have COVID-19 (64.2%), compared with those who did not (41.7%) (OR = 2.5; 95% CI = 1.1–5.8); prevalence was also higher among persons who reported sharing the same sleeping berth with a crewmember who had positive test results (65.6%), compared with those who did not (36.4%) (OR = 3.3; 95% CI = 1.8–6.1). Lower odds of infection were independently associated with self-report of wearing a face covering (55.8% versus 80.8%; OR = 0.3; 95% CI = 0.2–0.5), avoiding common areas (53.8% versus 67.5%; OR = 0.6; 95% CI = 0.4–0.9), and observing social distancing (54.7% versus 70.0%; OR = 0.5; 95% CI = 0.3–0.8), compared with service members who did not report these behaviors.

## Discussion

In this convenience sample of young, healthy U.S. service members experiencing close contact aboard an aircraft carrier, those with previous or current SARS-CoV-2 infection experienced mild illness overall, and nearly 20% were asymptomatic. Approximately one third of participants reported fever, myalgia, and chills and had higher odds of SARS-CoV-2 infection than did persons who reported cough and shortness of breath. Participants reporting anosmia (loss of sense of smell) or ageusia (loss of sense of taste) had 10 times the odds of having infection, compared with those who did not.

A study of adolescents and young adults with mild COVID-19 illness in China found rapid propagation of chains of transmission by asymptomatic persons (*6*). Reporting symptoms of anosmia and ageusia was common, and these symptoms are recognized in other respiratory viral infections as well. Acute anosmia was reported among one in seven COVID-19 patients in a South Korean study and was perceived to be an important sign of the disease (*7*). Others concluded that new onset anosmia should be considered SARS-CoV-2 infection until proven otherwise and recommended immediate isolation and confirmatory testing in persons with this symptom (*8*). Whereas anosmia or ageusia alone was predictive of COVID-19, absence of either of these symptoms should not be used to rule out SARS-CoV-2 infection.

Nearly two thirds of persons in this sample had positive ELISA test results, which indicate previous exposure to SARS-CoV-2. Among those who provided NP swab samples, approximately one third had positive real-time RT-PCR test results, some having recent symptom onset without evidence of having yet developed an antibody response. In another study, seroconversion among laboratory-confirmed COVID-19 patients was observed a median of 11 days after symptom onset for total antibodies and longer for more virus-specific antibodies, including neutralizing antibodies (*9*). The results from the current study reflect the intensity of exposure experienced by these participants and the recency of the outbreak at the time of specimen collection.

The shipboard environment presents substantial challenges for reducing viral transmission because of congregate living quarters and close working environments. The significant association of infection and male sex could reflect an association with berthing, which is separated by sex aboard the ship. Protective behaviors included wearing a face covering and maintaining physical distance. Multiple cruise ship outbreaks have documented undetected transmission of SARS-CoV-2 because of mild and asymptomatic infection (*10*). In outbreak investigations of younger crew members aboard cruise vessels, transmission was associated with working on the same deck and being within the same occupational group as persons with confirmed cases (*1*).

In this sample of intensely exposed subjects, assessed at a single point in time, results demonstrated that antibodies developed and that, at the time of specimen collection, many of these were neutralizing antibodies. Affinity maturation of antibodies is an important determinant for the outcome of viral infection. High-affinity antibodies can elicit neutralization by recognizing specific proteins on the surface of the virus, and these might be produced early or late in the course of viral infection. Approximately one half of the participants with positive ELISA results also had neutralizing antibodies, which indicate functional antibodies that would be expected to inhibit SARS-CoV-2 infection. This is a promising indicator of immunity, and in several participants, neutralizing antibodies were still detectable >40 days after symptom onset. Ongoing studies assessing the humoral antibody response over time will aid the interpretation of serologic results in an outbreak investigation such as this.

The findings in this report are subject to at least four limitations. First, the analysis was conducted on a convenience sample of persons who might have had a higher likelihood of exposure, and all information was based on self-report, raising the possibility of selection and recall biases. The sex and ethnic distribution of the participants was similar to that of all service members aboard the aircraft carrier, although survey participants were slightly older and of a slightly different racial distribution; therefore, they might not be a representative sample. Second, this analysis was limited by the lack of temporal data on previous positive test results for SARS-CoV-2, which complicates interpretation of the ELISA and microneutralization assays. Third, although the date of any symptom onset was collected, information on timing, duration, and severity of individual symptoms was not collected. Finally, the cross-sectional nature of these data might underestimate the eventual antibody response and neutralizing antibody activity among persons tested early in the course of their infections.

These results provide new indications of symptomatology of SARS-CoV-2 infections and serologic responses among a cohort of young U.S. adults living in a congregate environment and contribute to a better understanding of COVID-19 epidemiology in the U.S. military. The findings reinforce the importance of nonpharmaceutical interventions such as wearing a face covering, avoiding common areas, and observing social distancing to lower risk for infection in similar congregate living settings.

**Summary**

**What is already known about this topic?**

Information about COVID-19 among young adults is limited.

**What is added by this report?**

Among a convenience sample of 382 young adult U.S. service members aboard an aircraft carrier experiencing a COVID-19 outbreak, 60% had reactive antibodies, and 59% of those also had neutralizing antibodies at the time of specimen collection. One fifth of infected participants reported no symptoms. Preventive measures, such as using face coverings and observing social distancing, reduced risk for infection.

**What are the implications for public health practice?**

Young, healthy adults with COVID-19 might have mild or no symptoms; therefore, symptom-based surveillance might not detect all infections. Use of face coverings and other preventive measures could mitigate transmission. The presence of neutralizing antibodies among the majority is a promising indicator of at least short-term immunity.

## Footnotes

* Fever, chills, muscle pain, headache, sore throat, new taste or smell disorder.
